# Proteasome as a Molecular Target of Microcystin-LR

**DOI:** 10.3390/toxins7062221

**Published:** 2015-06-17

**Authors:** Zhu Zhu, Li Zhang, Guoqing Shi

**Affiliations:** School of Chemistry and Biological Engineering, University of Science and Technology Beijing, Beijing 100083, China; E-Mails: zhuzhu@ustb.edu.cn (Z.Z.); lizhang890125@163.com (L.Z.)

**Keywords:** microcystin-LR, proteasome trypsin-like activity, mechanism, molecular docking

## Abstract

Proteasome degrades proteins in eukaryotic cells. As such, the proteasome is crucial in cell cycle and function. This study proved that microcystin-LR (MC-LR), which is a toxic by-product of algal bloom, can target cellular proteasome and selectively inhibit proteasome trypsin-like (TL) activity. MC-LR at 1 nM can inhibit up to 54% of the purified 20S proteasome TL activity and 43% of the proteasome TL activity in the liver of the cyprinid rare minnow (*Gobiocypris rarus*). Protein degradation was retarded in GFP-CL1-transfected PC-3 cells because MC-LR inhibited the proteasome TL activity. Docking studies indicated that MC-LR blocked the active site of the proteasome β2 subunit; thus, the proteasome TL activity was inhibited. In conclusion, MC-LR can target proteasome, selectively inhibit proteasome TL activity, and retard protein degradation. This study may be used as a reference of future research on the toxic mechanism of MC-LR.

## 1. Introduction

The frequent occurrence of harmful algal bloom with exacerbating inland water eutrophication has become a global environmental problem [1]. Microalgal bloom often produces microcystins (MCs), which are hepatotoxins that may induce hepatic carcinoma development [2]. The general structure of MCs contains cyclo-(-d-Ala-l-X-erythro-β-methyl-d-isoAsp-l-Y-Adda-d-isoGlu-*N*-methyldehydro-Ala). Adda (3-amino-9-methoxy-2,6,8-trimethyl-10-phenyldeca-4,6-dienoic acid) is a unique beta amino acid that consists of 20 carbon atoms, which is responsible for the bioactivity of MCs [3]. More than 80 MC congeners had been identified because of X and Y variables [4,5]. MC-LR with the variable amino acids leucine (L) and arginine (R) is the most frequently investigated MC variant; MC-LR is, also one of the most potent hepatotxins [6]. Furthermore, MC-LR exposure adversely affects various animal organs and organ systems, such as liver, kidney, digestive tract, gonads, immune system, hypothalamic-pituitary system, and nervous system [3,7,8,9,10,11,12]. Well-documented toxic mechanisms for MC-LR are its inhibition to serine/threonine protein phosphatases (PPs) and induce oxidative stress in animal cells [13]. However, several enzymes, such as catalase [14], glutathione *S*-transferase [15], L-3-hydroxyacyl coenzyme A dehydrogenase [15], aldehyde dehydrogenase 2 [16], and ATP-synthase [17], were proved to interact with MC-LR and contribute to its toxicity or biotransformation. These new insights demonstrate the complexity of the interaction of MC-LR with animal cells.

The ubiquitin-proteasome system (UPS) degrades proteins in eukaryotic cells; this system is important in cell cycle and apoptosis [18,19]. The 26S proteasome is composed of a 20S core catalytic complex flanked on both sides by the 19S regulatory complexes [20]. The 20S core particle, which is a cylinder composed of four stacked rings, exerts minimal proteolytic activity in cellular environments [20]. The catalytic chamber of 20S core particle is formed by two inner β rings, each of which contains three active sites, namely, chymotrypsin-like (CT), trypsin-like (TL), and post-glutamyl peptide hydrolase-like (PGPH), which are located in the β5, β2, and β1 subunits, respectively [21]. Although the proteasome CT activity has been extensively investigated [22,23,24], the functions of proteasome TL activity in protein degradation and cell cycle are seldom explored. Saling [25] observed that the TL activity, not the CT activity, is involved in the binding of mouse spermatozoa to zonae pellucidae; this phenomenon indicates that a toxic mechanism is involved in the TL activity. The TL activity is also relatively higher than CT and PGPH activities in sperm extracts from several mammalian species, including hamster, mice, rats, cattle, rabbits, and humans [26]. Mirabella [27] demonstrated that inhibitors of proteasome TL sites selectively sensitize myeloma cells to bortezomib and carfilzomib, which are CT activity inhibitors.

Trypsin and TL protease hydrolyze peptide bonds with an amine group from amino acid residues of Arg and Lys; nevertheless, MC-LR cannot be degraded by protease at neutral pH [28]. Considering these findings, we hypothesized that MC-LR binds to trypsin and TL protease; thus, trypsin and TL protease activities are inhibited. If this hypothesis is true, then MC-LR exposure may retard protein degradation by the ubiquitin-proteasome pathway, disturb normal protein turnover in cells and induce toxic effects. To test this hypothesis, we investigated the effect of MC-LR on proteasome activity *in vitro* and *in vivo*.

## 2. Results and Discussion

### 2.1. MC-LR Selectively Inhibited Proteasome TL Activity

**Figure 1:**
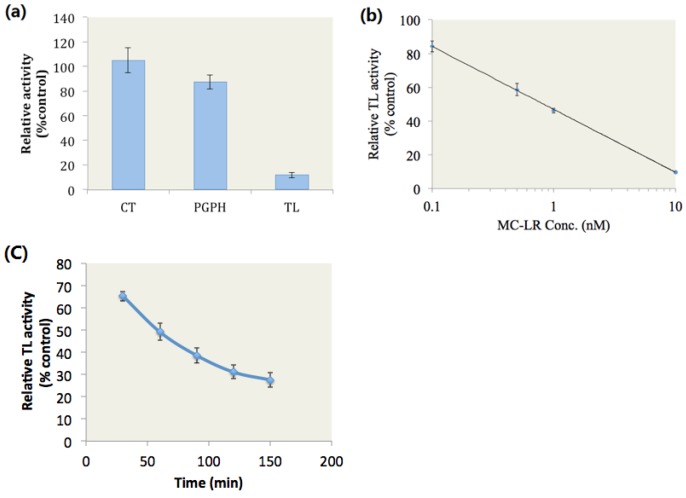
Inhibitory effects of MC-LR on proteasome activity. (**a**) MC-LR (10 nM) selectively inhibits proteasome TL activity. (**b**) MC-LR inhibits proteasome TL activity in a dose-dependent manner. (**c**) MC-LR (2 nM) inhibits proteasome TL activity in a time-dependent manner. (*n* = 3)

We hypothesized that MC-LR binds to the active site of the proteasome β2 subunit and then inhibits the proteasome TL activity. To test this hypothesis, we investigated the inhibitory effects of MC-LR on the proteasome CT, PGPH and TL activities of the purified 20S proteasome. The purified human 20S proteasome was incubated with the fluorogenic peptide substrates for proteasome CT, PGPH, and TL activities in the presence of 10 nM MC-LR or in the presence of methanol as solvent for 2 h at 37 °C. Fluorescence was then determined. Compared with methanol, 10 nM MC-LR inhibited 88.4% of the proteasome TL activity (Figure 1a). By contrast, MC-LR did not evidently affect the proteasome CT and PGPH activities.

The TL activity of the 20S proteasome was determined after the purified 20S proteasome was incubated with the fluorogenic peptide substrates and MC-LR at different concentrations for 2 h at 37 °C. The results showed that MC-LR with an IC50 of 0.8 nM dose-dependently inhibited the proteasome TL activity (Figure 1b).

MC-LR and proteasome TL activity were subjected to kinetic analysis by incubating the purified 20S proteasome with fluorogenic peptide substrates and 2 nM MC-LR for 30–150 min at 37 °C. Fluorescence was then determined. We observed that MC-LR time-dependently inhibited the proteasome TL activity (Figure 1c).

### 2.2. MC-LR Exposure Retarded the Degradation of Green Fluorescent Protein (GFP) by Proteasome

To examine whether or not MC-LR impedes cellular protein degradation, PC-3-GFP^u^ cells were treated with 0.1 μM MC-LR and 1 μM MG-132 for 24 h, respectively. PC-3-GFP^u^ cells are PC-3 cell lines that are stably transfected with the UPS reporter plasmid GFP^u^ [29]. The GFP^u^ gene consists of a short CL1 degron fused to the carboxyl-terminus of GFP. The short peptide ACKNWFSSLSHFVIHL encoded by CL1 is a degradation signal for UPS [30]; the GFP product is continuously degraded and maintained at very low levels under normal conditions. However, ubiquitinated GFPs accumulate when proteasome activity is inhibited, and this phenomenon can be observed through fluorescence microscopy [31]. The cellular uptake of microcystins (MCs) requires specific organic anion transporting polypeptides (OATPs) [32]. It has been reported that high OATP levels are expressed in castration-resistant prostate cancer (CRPC) [33], as a typical cell line of CRPC, the PC-3 cells has high susceptibility to uptake MC-LR.

**Figure 2:**
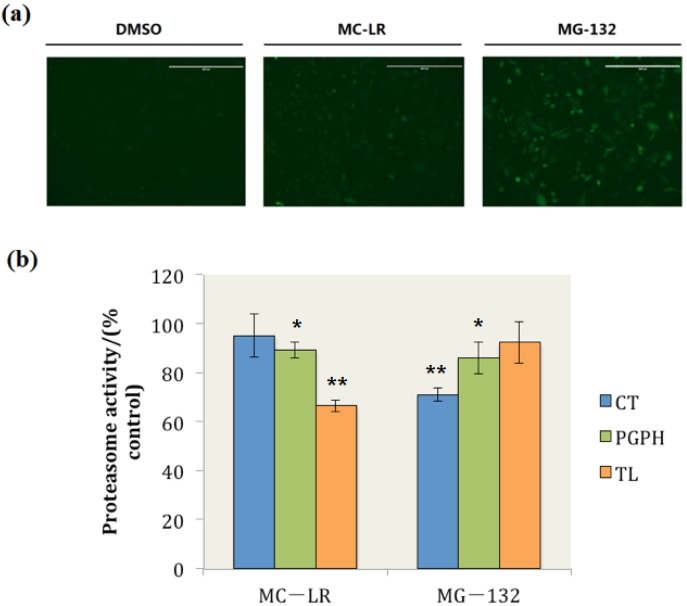
MC-LR exposure retards GFP^u^ degradation by proteasome and inhibits cellular proteasome TL activity. (**a**) PC-3-GFP^u^ cells treated with DMSO, 0.1 μM of MC-LR and 1 μM of MG-132 (as positive control), respectively. (Scale bar = 400 μm) (**b**) The proteasome activity in MC-LR and MG-132 treated cells. (*n* = 3, significances *vs.* the control: * *p* < 0.05, ** *p* < 0.01)

In this study, when the cells were treated with dimethyl sulphoxide (DMSO), the GFP was continuously degraded and maintained at very low levels, and the fluorescence of GFP could not be observed (Figure 2a DMSO). By contrast, GFP degradation was retarded and ubiquitinated GFP accumulated when the cells were exposed to MG-132, which is a well-documented proteasome inhibitor (Figure 2a MG-132). Ubiquitinated GFP also accumulated in MC-LR treated cells (Figure 2a MC-LR); this finding indicated that MC-LR inhibits the proteasome activity in PC-3-GFP^u^ cells and hinders the degradation of GFP by proteasome.

The proteasome CT, TL, and PGPH activities in whole cell extracts were also determined. The results showed that 34% of the proteasome TL activity was inhibited by MC-LR; by contrast, the proteasome PGPH activity was slightly inhibited by MC-LR, and the proteasome CT activity did not evidently change compared with those of the control group (Figure 2b). These results indicated that MC-LR could inhibit the cellular proteasome TL activity and prevent the cellular degradation through proteasome.

### 2.3. MC-LR Inhibited the Proteasome TL Activity in the Liver of Gobiocypris rarus

**Figure 3:**
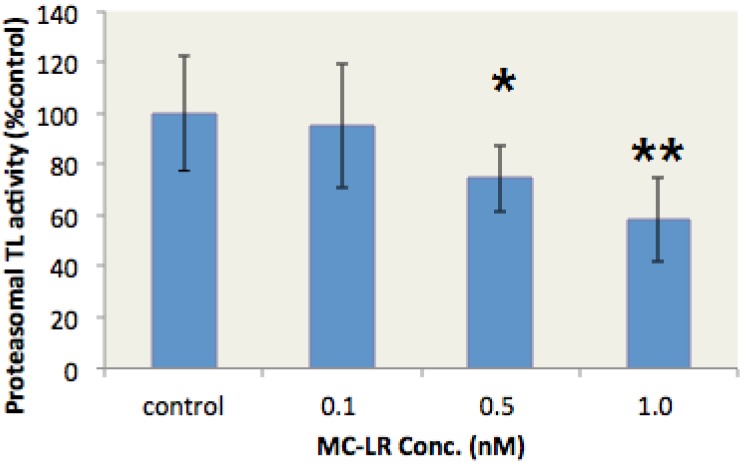
MC-LR inhibits the proteasome TL activity in the liver of *G. rarus*. (*n* = 5, significances *vs.* the control: * *p* < 0.05, ** *p* < 0.01)

*G. rarus* is an emerging fish model in aquatic toxicology in China [34]. This species is sensitive to environmental endocrine disruptors [35]. In our study, *G. rarus* individuals were exposed to different MC-LR concentrations for two days to verify whether MC-LR inhibits the proteasome TL activity *in vivo*. The proteasome TL activity in the liver of *G. rarus* was subsequently determined. The proteasome TL activity in the liver of *G. rarus* was dose-dependently inhibited by MC-LR (Figure 3). Moreover, 1 nM MC-LR resulted in a 43% inhibition rate. These data indicated that MC-LR could inhibit proteasome TL activity *in vivo*.

### 2.4. Docking Studies Indicated that MC-LR could Block the Active Site of the Proteasome β2 Subunit but not the Active Site of β1 and β5 Subunits

**Figure 4:**
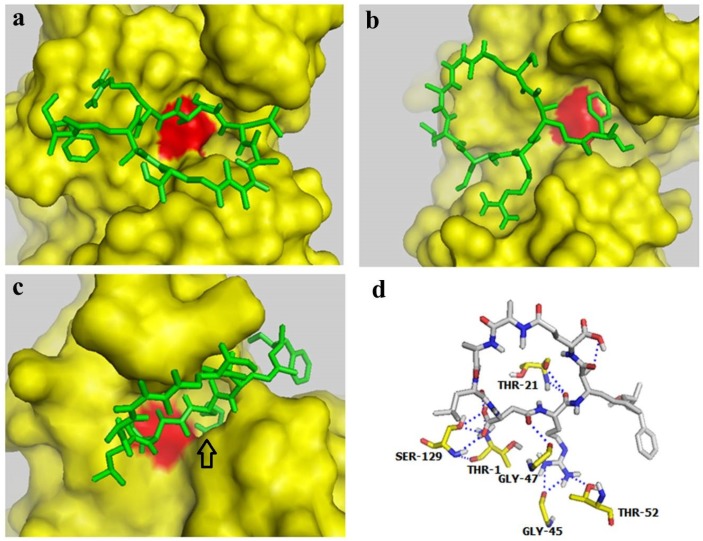
Docking studies for interaction between MC-LR and proteasome subunits. (**a**–**c**) Docking models for binding of MC-LR to the proteasome subunits β1, β5, and β2, respectively. (**d**) Docking model for interaction between MC-LR and the proteasome β2 subunit.

Autodock 4.0 software is a free docking tool designed to predict the manner by which small molecules bind to a receptor of a known 3D structure. This tool has been successfully utilized in docking analyses of several proteasome inhibitors [36,37]. In the present study, Autodock 4.0 software was used to investigate the interaction of MC-LR with the β1, β5, and β2 subunits of the 20S proteasome. The conformations with the lowest docking free energy of MC-LR on the β1, β5, and β2 subunits are shown in Figure 4a–c, respectively. In Figure 4a,b, MC-LR did not completely fill the cavity with catalytic residue (marked by red) on the β1 and β5 subunits. This result indicated that MC-LR could not block the active site of the β1 and β5 subunits. MC-LR completely filled the cavity of the β2 active site; the side chain of the Arg residue in MC-LR was also deeply inserted into the P1 package of the β2 subunit (indicated by an arrow in Figure 4c). The binding of MC-LR and the β2 subunit could be further enhanced by the hydrogen bond between MC-LR and the amino acid residues of THR-1, THR-21, GLY-45, GLY-47, THR-52, and SER-129 on the β2 subunit (Figure 4d). No covalent bond was observed between MC-LR and the β1, β5, and β2 subunits of the 20S proteasome. These results indicated that MC-LR selectively inhibits the proteasome TL activity by tightly binding to the proteasome β2 active site cavity. This phenomenon elucidates the different inhibitory effects of MC-LR on proteasome PGPH, CT, and TL activities.

## 3. Materials and Methods

### 3.1. Materials

MC-LR was purchased from Taiwan Algal Science Inc. (Yangmei City, Taoyuan County, Taiwan). MC-LR was diluted with DMSO to prepare 100 μM stock solution, which was stored at −20 °C. The purified human 20S proteasome, and the fluorogenic peptide substrates Suc-LLVY-AMC (for CT activity), Bz-VGR-AMC (for TL activity), and Z-LLE-AMC (for PGPH activity) were obtained from Enzo Life Sciences Inc. (Farmingdale, NY, USA). Ultrafiltration membrane (500 kD NMWL) was purchased from Millipore (Billerica, MA, USA).

### 3.2. MC-LR Inhibition of Purified 20S Proteasome Activity

For the determination of proteasomal TL, CT, and PGPH activity, The purified human 20S proteasome (0.02 μg) was incubated in 100 μL of assay buffer (25 mM Tris-HCl, pH 7.5) with MC-LR and 10 μM of the specific fluorogenic peptide substrates at 37 °C for 2 h respectively. Fluorescence was determined using a Victor2 multilabel reader microplate fluorometer (Perkin Elmer, Waltham, MA, USA) with a 380 nm excitation filter and a 460 nm emission filter.

### 3.3. MC-LR Exposure to PC-3 Cells with UPS Reporter

PC-3 cells were stably transfected with the UPS reporter plasmid GFP^u^ as previously described [29]. The GFP^u^ gene consists of a short CL1 degron fused to the carboxyl-terminus of GFP. The short peptide ACKNWFSSLSHFVIHL encoded by CL1 is a degradation signal for UPS [30]. A clonal cell line stably expressing GFP^u^ was isolated and named as PC-3-GFP^u^. The GFP^u^ was continuously degraded and maintained at very low levels under normal conditions. GFP^u^ accumulated after proteasome was inhibited. PC-3-GFP^u^ cells were cultured in an F12 medium supplemented with 10% fetal bovine serum and 1% penicillin/streptomycin under a humidified atmosphere of 5% CO2. Cell cultures were treated with 0.1 μM MC-LR (dissolved in DMSO) for 24 h and then imaged through digital fluorescence microscopy (EVOS-f1, AMG, Mill Creek, WA, USA). After photos were taken, the proteasome activity in whole cell extracts was determined as previously described [38]. The cells were treated with DMSO and 1 μM of MG-132 (a well-documented proteasome inhibitor) as negative and positive controls, respectively.

### 3.4. MC-LR Inhibition of the Proteasome Activities in G. rarus

Eighty *G. rarus* individuals were randomly divided into four groups (*n* = 20 per group). The groups were exposed to different MC-LR concentrations in fresh water for two days and then dissected. The proteasome TL activity in the liver of the fish in each group was determined, as described in a previous study [39] with slight modifications. The fish in each group were randomly distributed into five subgroups (*n* = 4 per subgroup). The liver samples from each subgroup were mixed, homogenized in extraction buffer (25 mM Tris-HCl, pH 7.4) with 0.25 mM sucrose, and then centrifuged at 12,000 rev/min for 30 min. The supernatant was collected, and the volume of the supernatant was increased by 20× in washing buffer (25 mM Tris-HCl, pH 7.4) with 20% glycerol and 2 mM ATP. The diluted sample was concentrated on a 500 kDa cut-off membrane to obtain the original volume. This procedure was repeated four times by using freshly prepared buffer in each time. All of these procedures were conducted at 4 °C. The protein concentrations of the four groups were determined using the Bradford method and were adjusted to the same level. An aliquot (2 μL) of the sample solution was incubated with Bz-VGR-AMC (1 mM) in the assay buffer (25 mM Tris-HCl, pH 7.4) at 37 °C for 2 h. The cleaved fluorescent products were determined at 380 nm excitation and 460 nm emission using a fluorescence plate reader.

### 3.5. In silico Modeling of MC-LR Binding to Proteasome Subunits

The binding of MC-LR to proteasome subunits was analyzed *in silico* using AutoDock 4.0 software (from the Scripps Research Institute, La Jolla, CA, USA), similar as described previously [36]. We initially refined the MC-LR molecular by performing an optimized geometry calculation of the saved Protein Data Bank (PDB) files; this procedure was conducted using the conversion filters in CAChe software V6.1.10 (Fujitsu, Fairfield, NJ, USA). The output PDB files were imported into AutoDock 4.0 software for the docking analysis to the proteasome subunits. The crystal structures of the β1, β2, and β5 subunits of the 20S proteasome were obtained from the yeast 20S proteasome (PDB 1JD2), which is similar to the human proteasome [40]. The docking space was limited to a 40 × 40 × 40 Å box centered on the catalytic *N*­terminal threonine prepared as an energy­scoring grid. AutoDock outputs were visualized and analyzed with PyMOL software (Schrödinger, New York, NY, USA) [36].

### 3.6. Statistical Analysis

Each experiment was repeated thrice. Data were indicated as mean ± standard deviation (±*s*). Student *t*-test was performed to evaluate the difference between treated and control groups.

## 4. Conclusions

In this study, we confirmed that MC-LR could selectively inhibit the proteasome TL activity in a dose- and time-depended manner by incubating the purified 20S proteasome with MC-LR. When GFP-CL1-transfected PC-3 cells were exposed to MC-LR, GFP proteins accumulated, and the cellular proteasome TL activity was inhibited. In animal experiments, the environmental level of MC-LR could inhibit the proteasome TL activity in the liver of the *G. rarus*. The results of docking studies indicated that MC-LR blocked the active site of the proteasome β2 subunit; thus, the proteasome TL activity was inhibited. In conclusion, MC-LR can target proteasome, selectively inhibit proteasome TL activity, and retard protein degradation. This study may be used as a reference for future research on the toxic mechanism of MC-LR. As one of the major toxic effects of MC-LR involves the induction of reactive oxidative species (ROS), the inhibitory effect of MC-LR on the proteasome TL activity may enhance the toxic effects of ROS because the UPS is the major pathway implicated in the degradation of ROS-damaged proteins.
